# Barriers to the implementation of electronic medical records in Northwest Ethiopia

**DOI:** 10.4102/hsag.v30i0.3010

**Published:** 2025-09-20

**Authors:** Asmamaw K. Tsehay, Kholofelo L. Matlhaba

**Affiliations:** 1 Department of Public Health, College of Medicine and Health Sciences, Bahir Dar University, Bahir Dar, Ethiopia; 2 Department of Health Studies, College of Human Sciences, University of South Africa, Pretoria, South Africa

**Keywords:** electronic medical records, implementation, barriers, Digital Health, Ethiopia

## Abstract

**Background:**

Although electronic medical records (EMRs) have a significant impact on the health system, their development is in its infancy in Ethiopia because of factors that hinder the implementation of the system.

**Aim:**

This study aims to explore the barriers to the implementation of EMRs.

**Setting:**

The study setting was in Northwest Ethiopia.

**Methods:**

This study employed a phenomenological qualitative research design to explore the lived experiences of health workers. Purposive sampling was used to recruit eight participants from five different health facilities, each holding diverse professional roles. Data were collected through in-depth interviews, which were audio-recorded and subsequently transcribed verbatim. The qualitative data were managed and analysed using ATLAS.ti software, following a thematic approach. The analysis adhered to the seven-step framework outlined in Colaizzi’s method.

**Results:**

A total of 24 codes, 10 subthemes and 4 major themes emerged from the analysis. Three of the major themes reflected challenges associated with EMRs, while one highlighted perceived benefits. The four major themes identified were software-related factors, system-related factors, institutional operational factors and benefits of EMRs.

**Conclusion:**

From the users’ perspective, the study finally comprehended that lack of EMRs training, lack of comprehensiveness of the system, a lack of interoperability, power interruption, system slowdowns and absence of incentives and rewards were barriers to the implementation of the EMRs system.

**Contribution:**

This study helps to understand the key barriers to the implementation of EMRs, offering insights for addressing these challenges in future EMR adoption efforts.

## Introduction

New technologies are continuously being adopted in health services (Rahimi et al. 2018:605). Nowadays, digital health technologies, like electronic medical records (EMRs), are being broadly introduced in health care, and they help to strengthen and transform the health system (Janssen et al. 2021:2). An EMR is a digital system used to manage and store a patient’s health information within a medical facility. Its main goal is to integrate health care data to enhance the quality of care. Electronic medical record systems are built to accurately store and track patient information over time, reflecting their health status through records such as personal details and demographics, medical history, diagnostic data including lab test results, and surgical information (Yip et al. 2019).

Currently, EMR systems are found in everyday practices among nursing and medical health care workers in the primary and acute care (Lloyd et al. 2021). Implementing EMRs in developing countries improves health care services, justifying the cost (Adetoyi & Raji 2020). Sub-Saharan Africa, home to 12% of the global population, bears 27% of the world’s disease burden. Despite this, it lags in health information technology (HIT), which is crucial for improving patient care (Femi Odekunle, Srinivasan & Odekunle 2018). These countries, however, have struggled to initiate large-scale EMR systems. In addition, keeping patient records secure is one of the key challenges in the implementation of electronic health records (EHRs). There are concerns related to the misuse of the database and threat to cyber-security (Wadhwa 2020).

In Ethiopia, EMR is becoming essential for modern patient care. A study at St. Paul’s and Ayder hospitals found that implementation fell short because of logistical challenges, poor internet, inadequate training and limited management engagement. St. Paul’s staff cited poor project management, weak Information and Communication Technology (ICT) infrastructure and lack of training as key barriers (Bisrat et al. 2021). Engaging health care workers in EMR design and training is key to successful implementation. Their perception of improved EMR features helps address usability challenges and enhance patient management. Continuous evaluation and support are essential for sustained use (Janssen et al. 2021:2–8). In low-resource settings, EMR implementers should pay attention to the four issues, namely, technical, human, process and organisational factors, to adopt the system and sustain EMRs’ use in the health sectors (Ngugi, Were & Babic 2018:1).

Designing a user-friendly interface is challenging, as a poor design can reduce efficiency, lower care quality and risk patient safety. Doctors also see EHR adoption as a barrier because of the extra time spent on data entry instead of patient care (Wadhwa 2020).

In addition, there was a significant relationship between the adoption of EMR and techno-organisational factors, and EMR-related infrastructure (Semo Isemeck et al. 2019). Weak internet connectivity and unstable power supply were key technological challenges, while limited financial resources, insufficient training support from hospital management, inadequate technical expertise and lack of consistent legal enforcement hindered EHR adoption (Semo Isemeck et al. 2019).

For the effective implementation and sustainability of the EMR system, the health care facilities should determine the barriers such as lack of technical support, workload and the ability of the health care workers to be familiarised with the EMR system (Janssen et al. 2021:5). Hence, the aim of this study is to explore the barriers of EMRs in Northwest Ethiopia to give insight about the case in the resource-limited areas.

## Research methods and design

### Study design

This study employed a phenomenological qualitative research design to explore barriers to EMR implementation based on health care workers’ experiences (Jung et al. 2021). Specifically, a descriptive phenomenological approach was used to capture participants’ lived experiences while minimising researcher bias. This design facilitated an in-depth understanding of the essence of participants’ experiences with EMR use.

Reflexivity plays a critical role in acknowledging the researcher’s positionality and potential influences on the research process. As researchers with a background in public health, our prior experience may have shaped our understanding of the challenges and opportunities in EMR adoption. Being familiar with both the local health care context and technological barriers, we are aware of the potential biases this may introduce in interpreting the data. By reflecting on these factors, the researchers strived to mitigate any biases by engaging in regular reflexive journaling and peer debriefing.

### Setting

This study was conducted in health care facilities in Bahir Dar City, Northwest Ethiopia. The city has seven hospitals, ten government-owned health centres and multiple private clinics. However, only five health care facilities that had implemented EMRs were included. Among the selected health facilities, two were government-owned health care facilities and three were private health facilities. All of them are situated in the urban area of Bahir Dar. These facilities were selected based on the extent of EMR implementation, level of patient volume and facility type to ensure diverse perspectives.

### Study population and sampling strategy

The study population was all health care workers who are currently practising on EMRs at health care facilities in Bahir Dar City, Northwest Ethiopia. This includes physicians, health officers, nurses, midwifery, laboratory professionals and pharmacy professionals, information system officers and medical data clerks who are working on EMRs. Participants were selected from the five health care facilities, and participants who had 2 years and above experience on EMRs were included in the study. The sample size was determined by data saturation, where no new information was discovered after some point. It was noticed during data collection by systematically comparing new data with previously collected interviews. When subsequent data no longer introduce significant new themes or insights, and patterns begin to repeat, it indicates that saturation was reached.

Purposive sampling was used to recruit health care workers where units are chosen because they meet the requirements of the sample or deliberately. Health facility administrators and the researchers’ judgement played a crucial role in this purposive sampling technique to discover and choose the health care professionals in various positions who can offer the most information to meet the study’s objectives (Creswell 2018). Of the twelve health care workers approached, eight consented to participate and four declined, citing workload pressures and lack of interest. The researchers’ prior professional and academic engagement with the facilities and health system enabled smoother interaction with both stakeholders and participants.

### Data collection

As part of the research process, creating an initial set of interview questions necessitates careful consideration of what to ask and how to ask it. The format of the interview questions is determined by the expertise and background knowledge of the researchers (Roberts 2020). The research instruments were designed thematically to ensure a structured yet flexible exploration of health care workers’ experience on EMR. In the semi-structured interview questions, the grand interview question is drawn from the main objective of the study and probing questions were developed to obtain detailed information on the problem. Questions were used to explore the overall experience with using the EMR system, along with probing questions such as: ‘Have you encountered any problems while using the EMR system in your daily practice?’ and ‘What barriers or challenges have you faced in implementing or using the EMR system at your health facility?’

The data were collected by in-depth interview method, where information was obtained from experts on the subject at hand. The interview was audio-recorded, facilitated by the data collector using a semi-structured interview guide. In addition, to make all the necessary preparations before starting data collection, the interviewer considered not to disturb the setting, avoid exploiting the participant and inform the purpose of the study (Creswell 2018). A convenient time and place for the in-depth interviews was arranged through discussions with the facility leader. The interviews were then conducted in a private room within the facility after working hours in the Amharic language, which is native to the setting. Informed consent was obtained from all participants prior to the start of the interview.

The recordings were transcribed verbatim into Amharic and then translated into English. Each transcript was then stored in Microsoft word, and quotes were extracted and coded using a data analysis software (ATLAS.ti).

## Data analysis

Data analysis followed Colaizzi’s seven-step method (Morrow et al. 2015) to ensure a systematic and rigorous approach. The process began with familiarisation, where transcripts were read multiple times to achieve immersion in the data. Next, significant statements related to EMR use were identified and extracted. These statements were then analysed to formulate meanings, allowing for a deeper interpretation of participants’ experiences. Following this, themes were clustered by organising formulated meanings into broader categories. An exhaustive description was then developed to provide a comprehensive narrative of the findings. The emerging themes were further refined to establish a fundamental structure, ensuring clarity and coherence. Finally, the findings were interpreted by synthesising insights and drawing meaningful conclusions.

### Techniques to enhance trustworthiness

The trustworthiness of the data was ensured throughout the research process, from data collection to analysis. The researchers employed strategies such as peer debriefing, triangulation and member checking to enhance trustworthiness. Member checking was conducted by sharing summaries of the findings with research participants. To ensure transferability, the researchers provided comprehensive contextual information and confirmed that the findings were grounded in data obtained from study participants, rather than influenced by personal interpretations or biases.

### Ethical considerations

The College Research Ethics Committee (CREC) at the University of South Africa’s College of Human Sciences (CHS) granted ethical approval on 23 November 2023 for the study (CREC Reference: 18033342_CREC_CHS_2023). Permission to access the city administration health office, which oversees the health care facilities under study, and to collect data from the five targeted health care facilities in Bahir Dar province was obtained from the Amhara Public Health Institute. Only participants who provided signed informed consent were included in the study. Confidentiality and anonymity were ensured, as the interviews were conducted in secure settings chosen by the participants. During the interviews, participants’ real names were not used. Furthermore, a confidentiality agreement was established with both the statistician and the research assistant to ensure the privacy and security of the data and research.

## Results

### Description of study participants

The study participants were health care workers from five health facilities in Bahir Dar City, Northwest Ethiopia. In-depth interview was conducted with the participants until information saturation was reached. The age range of the participants was from 28 years to 38 years. Among the study participants, five were female participants and three were male participants, and their professions were multidisciplinary (Table 1).

**TABLE 1:** Description of participants.

Participants	Health facility	Age (years)	Sex	Profession
P1	Health facility 1	32	Female	Nurse
P2	Health facility 1	28	Female	Nurse
P3	Health facility 2	30	Male	Medical doctor
P4	Health facility 2	35	Female	Medical records professional
P5	Health facility 3	31	Female	Medical doctor
P6	Health facility 3	34	Male	System administrator
P7	Health facility 4	38	Female	Medical laboratory professional
P8	Health facility 5	26	Male	Maternal and child health officer

## Themes and codes

Twenty-four codes, ten subthemes and four major themes emerged from this study. Among these major themes, three were related to the challenges of EMR’ implementation, while one reflected the benefits of EMRs as reported by the participants. The major themes identified in the study are software-related factors, system-related factors, institutional operation-related factors and benefits of EMRs. These themes, along with their corresponding subthemes and codes, are presented in Table 2.

**TABLE 2:** Codes, subthemes and themes emerged from the qualitative study.

Major themes	Subthemes	Codes
1. Software-related factors	1.1. Interoperability	Lack of interoperability
	1.2. Comprehensiveness	Lack of clinical procedures and modules
		Incomplete point of care terminologies
		Incomplete diagnostic names and drug names
		Lack of comprehensiveness
	1.3. Software version	Older version
2. System-related factors	2.1. System problems	System fluctuation
		Delay in medication order
		System failure
		System slowdowns
		Delay in diagnostic result
	2.2. Hardware	Low server memory
3. Institution operation-related factors	3.1. Human resource	Shortage of trained human resources
		Shortage of human resource
	3.2. Infrastructure	Electric power interruption
	3.3. Responsibility	Run by funded projects
	3.4. Motivation and support	Low incentives and rewards
		Lack of trainings on EMRs
		Lack of integration
4. Benefits of EMR	4.1. Benefits of EMR	Easy information retrieval
		Save time and space
		Reduce duplication of MRN
		Help to retrieve diagnostic results
		Save time and space

EMR, electronic medical record; MRN, medical record number.

### Theme 1. Software-related factors

This theme encompassed the subthemes of interoperability, EMR comprehensiveness and software version. Participants highlighted several software-related challenges, ranging from limited content coverage and outdated software versions to poor user interface design and a lack of interoperability with other systems. These issues were seen as critical barriers affecting both the usability and acceptability of EMRs, ultimately influencing health care providers’ willingness and ability to use the system effectively.

#### Subtheme 1.1. Interoperability

One of the obstacles to the effective implementation of EMRs was the lack of interoperability. It was difficult to share data if health care workers did not use the same words or standard terminology. This may force the practitioners to put data in different places in the EMR. It is clear that, if we do not reach a consensus on the format of medical record transmission, we will put records in the wrong place.

Participants explained that the EMR system did not have the ability to communicate and exchange data with other systems from other health facilities. Hence, data integration and interoperability continue to be the most difficult challenges in health information systems. Here, the lack of terminology and structural standards, or implementation of the standards, contributed to the challenges. Participants expected the EMRs to connect different health facilities and services and to ease the exchange of information. However, many of the participants felt that this was not fulfilled. This is because different health facilities have implemented EMRs with different infostructures, which hindered interoperability.

In support of this, some participants said:

‘The other challenge I have seen is the system [*EMR*] only works within the vicinity of the health facility, so we cannot access the client’s medical history from another health institution …’ (Age 30, HF2, Male, MD, P3)

As the EMR is implemented with a local area network, it was not integrated with other health facilities. This hinders access to patient information from any location other than the facility, one participant stated:

‘I have learnt that the EMR system in our health facility is not internet based, so if the patients are to go to any other health facility in Bahir Dar City, the healthcare provider would not be able to access their information. Physicians cannot be able to know the patients progress from another facility and how they were being managed and the treatment they have had before.’ (Age 34, Female, HF2, Medical Record P, P4)

#### Subtheme 1.2. Comprehensiveness

Study participants recognised the benefits of EMRs, highlighting their impact on the efficiency of the health care service. And, to have an effective implementation of EMRs, the system is expected to be comprehensive, which in reality is one of the challenges to adopt EMR. In this regard, participants described that medical terminologies, diagnostic names and drug names were not found in the system, according to EMRs international standards.

Most of the participants supported these subthemes:

‘[*A*]nd there are drugs that I couldn’t find on this system with their scientific name. Also, I cannot access and get various manuals and clinical procedures or instructions from this system.’ (Age 32, Female, HF1, Nurse, P1)

‘This electronic medical record contains medical terminology, but not complete, meaning there are disease or diagnostic terms that are not found in the drop-down box while entering patient information and this should be considered during system upgrade.’ (Age 30, HF2, Male, MD, P3)

‘The problem I found in the system is that some diagnosis and drug names for prescription are not available in the electronic medical record system, which we are forced to write or to use manual medical record.’ (Age 31, Female, HF3, MD, P5)

‘[*B*]ut the main challenges are there are some clinical procedures and service data elements that are not found in the system …’ (Age 26, HF5, Male, MCH, P8)

#### Subtheme 1.3. Software version

Sometimes, even if the health facilities use EMRs from a similar vendor or the same product, they may use different versions of the product. This makes the job of exchanging data on an individual patient or a population challenging. In addition to this, participants described that one of the challenges of using EMRs was the software being outdated, which will not match today’s technology.

One participant discloses this as follows:

‘I have heard from experts that the system should be upgraded in order to improve the system performance and increase the speed. So, I wish if the stakeholders can understand the issue and collaborate to introduce a new EMR system or upgrade the existing one.’ (Age 30, HF2, Male, MD, P3)

### Theme 2: System-related factors

This major theme consists of categories or subthemes that included system problems and hardware problems. Electronic medical record system is a computerised medical information system that collects, stores and displays patient information. Electronic medical record systems in health facility settings provide clinical, communicational and administrative functionalities. So, there are challenges from conceptualisation to the implementation of the system that hinder the effectiveness and acceptance of EMRs.

#### Subtheme 2.1. System problems

As stated by the participants, networking problems and inadequate computer hardware remain the primary causes linked with issues like system interruption, delayed communication, system configuration, network installation and server memory.

Problems related to the system were system fluctuations, delay in medication order, system failure, diagnosis result delay and system slowdowns.

**System fluctuation:** In a computing system, there were clients or servers, including hardware, software and peripheral devices. The client or workstation is any computer system that a user interacts with directly (like desktop, laptop computers and workstations). Servers are computers or machines that receive, store, retrieve and communicate data between two workstations (client computers). So, the communication between two or more client devices in the EMR system is hindered by the system fluctuation.

A participant indicated this as:

‘Ohhh there are many challenges when using electronic medical records. And, one of them is system fluctuation. This incident [*system fluctuation*] were seen in most of the outpatient and inpatient rooms in case of weak network strength when the number of client computers reach beyond the capacity …’ (Age 32, Female, HF1, Nurse, P1)

**Delay in diagnostic result and medication order:** Sometimes, there is a report of delayed communication between clinical units caused by a system problem. Messages sent from the outpatient department may not reach the lab unit or the pharmacy department. This causes work overload at one unit of the health service.

This was indicated by the participants as follows:

‘Laboratory and radiology results may not arrive soon after submission from the department, and also prescriptions may not arrive at the pharmacy by the time you send them. This disrupts the workflow and the health system in the facility.’ (Age 28, Female, HF1, Nurse, P2)

Most health care providers use EMRs to prescribe drugs beyond capturing the patient information. However, there were delays in communication because of a system problem.

A nurse from facility one indicated this as:

‘… Sometimes a medication order may take more than 20 minutes to arrive to the pharmacy. I think the reason for the delay is a problem related to the EMR system. Moreover, the occurrence of power outages has a major impact and worsens the problem.’ (Age 32, Female, HF1, Nurse, P1)

**System slowdowns:** System slowdowns can occur because of poor network systems and inadequate power supply to the server and client devices. This problem disappoints most health care providers who frequently wait for the system to be active or responsive. Sometimes, practitioners are forced to use the paper medical record until the electronic system is fully restored.

In support of this, two participants stated that:

‘… It is no wonder that EMR systems provide a solution for multifaceted health information problems; but system-related slowdowns forced us to keep the paper-based systems with the EMRs parallelly. So, what we would do is to go back into the system from paper records after power downtimes. Or we re-enter the information from paper backup to the EMR.’ (Age 28, Female, HF1, Nurse, P2)

‘Though there was turnaround time set for the laboratory result to be dispatched, the lab result may not reach to the sender as soon as we have submitted and at the same time lab orders from OPDs and IPDs will not arrive soon to our lab. This delay causes disappointment among clients.’ (Age 38, Female, HF4, Medical Laboratory, P7)

#### Subtheme 2.2. Hardware

Although the term ‘computer hardware’ includes all physical components of a computer system, this subtheme focused on the server memory that the participants described as a barrier to use the EMR.

**Low server memory:** The servers and client computers have two different types of computing memory. It is believed that servers generally have more and faster central processing units and may also have larger memory sizes than the clients that interact with the server. There are two forms of computer memory, namely, Random Access Memory (RAM), which allows a computer to hold information and process it, and hard drives (storage), which are fixed memory, similar to long-term memory in people. So, participants learnt that most problems for the system to become slow and unresponsive arise from this. This would be the reason for patients to wait for a longer time and to raise complaints about the service.

One participant described it as:

‘[*T*]he other things we are having trouble with is related to the memory of the server. The server should have the capacity to handle the database and control the client computers, which in reality is not. This may cause a longer time lag to open the electronic medical record system at the point of care.’ (Age 34, HF3, Male, P6, System admin)

### Theme 3: Health facility operation-related factors

Most of these factors were expected to be managed by the health facility. The key challenges described in this category are human resource, infrastructure, responsibility, motivation and support from the health facility (Table 2).

#### Subtheme 3.1. Human resource

Human resource issues were strongly related to operational concerns. Health facilities in Bahir Dar City have a high staff turnover rate. It was challenging to provide incentives for EMR use, and the goals of the leaders do not always coincide with those of their staff. The commitment to leading the EMR was not always carried over by the new managers. And, there was a sense of missing operational continuity, particularly with regard to EMR accountabilities and responsibilities.

Participants found that to move away from paper-based medical records, the health facility management should have a stronger sense of purpose and dedication, while acknowledging that any system change will strain staff relations. It was frequently noticed how crucial clear-cut and constant leadership support is. Staff members would be less disturbed by incidents related to the EMR when they were informed in advance and when there was a trained experienced staff member. Management changes and the absence of well-trained system personnel, either on reserve or when needed, remain a challenge.

Participants explained this as follows:

‘[*G*]reat if the concerned party could come up with a non-disruptive solution and have an EMRs troubleshooting tutorial. Indeed, to promote EMRs utilization in our facility administrators should think of about incentives and rewards to us [*health professionals working on the EMRs*] … incentives and rewards would also motivate us. For me, this will not be a short time activity …’ (Age 28, Female, HF1, Nurse, P2)

‘The problem is worsened by the absence of a reserve EMR system support professional when the one in place who oversees the electronic medical record system leaves for meetings or training. There are times when a system is turned off for a day long due to the absence of the system administrator.’ (Age 28, Female, HF1, Nurse, P2)

#### Subtheme 3.2. Infrastructure

Electric power interruption: Unreliable electricity has proven to be a significant obstacle to the success of EMR transitions. In the health facilities in Bahir Dar City, power outages are frequent and may last up to 12 hours. Different Internet services may have unpredictable outages for different periods of time. Data transport speeds were unstable and may cause the EMR to lag so much that screens could take up to 20 min to load. And, updating a central data repository or server was one participant’s suggestion. Parallel paper-based systems had to be maintained because of system-related slowdowns, and some health facilities created the procedures for re-entering data from paper backups in the event of power outages:

‘I observed a disconnection problem on the system. In all outpatient departments, at some point the system stacks and you cannot be able to work on the user interface, this was mainly after power interruption.’ (Age 28, Female, HF1, Nurse, P2)

‘The challenge in our work is during a power outage, which forces us to wait 20 to 30 minutes for the electronic medical record system to restart. During that time patient complaints become unmanageable. And sometimes we are forced to work on manual records. The health facility has an electric generator, but it doesn’t completely solve the problem …’ (Age 30, HF2, Male, MD, P3)

Another participant also described how the problem persisted after power resumed, for some time until the system restored and the user interface opened (a higher system downtime), and how this problem was disappointing to the patients:

‘[*D*]uring power outage that it takes more than an hour the system to start working after power turned back. Due to this, the patients get into quarrels with us [*healthcare workers*], because they think that the system will start as soon as the electric power turns on.’ (Age 35, Female, HF2, Medical Record P, P4)

#### Subtheme 3.3. Responsibility

**Run by funded projects:** The cost of implementing an EHR was a major barrier for many hospitals and providers. Therefore, funding organisations in collaboration with the Ministry of Health are engaged in piloting this programme in a few health facilities. In this context, the projects assume that the programme could be sustained even after the funding organisation’s project ends. However, most EMR systems introduced by non-governmental organisations will face difficulty continuing, as most of the health facilities were not ready to take the responsibility when the project ends.

This was well explained by the participant:

‘… However, most of the support and assistance now comes from non-governmental institutions and donor funded projects; This does not ensure continuity, so government engagement must be substantial.’ (Age 34, HF3, Male, P6, System admin)

#### Subtheme 3.4. Motivation and support

**Low incentives and rewards:** Participants brought up the topic of financial and non-financial incentives for EMR adoption, although their opinions were divided. There were many who believed that those who put in more time and effort ought to be rewarded financially or with a certification of some kind. Others saw that financial rewards could not last. However, participants emphasised the need for acknowledgement and reward for celebrating implementation achievements differently like certification or financial reward.

One participant stressed this as:

‘… Additionally, the work we do and the payment we get are not commensurate. I can assure you that if there were a reward scheme to motivate care providers working on EMR or based on their performance, the program will get a high attention.’ (Age 32, Female, HF1, Nurse, P1)

Lack of training on electronic medical records: The use of EHRs requires staff training, which is essential for system uptake and compliance. System adoption can be aided by identifying highly motivated health care workers who will serve as superusers (system admin) and receive training on the system before it goes live. Being trained by a peer who speaks the same practice language makes many health care providers feel more comfortable.

Participants suggested that training is mandatory for both superusers and health care providers and helps to retain information and knowledge about the EMRs:

‘Upgrading the system, training and professional help is needed to address this system disruption; not only for the health care practitioner, but also for the body that controls the system.’ (Age 32, Female, HF1, Nurse, P1)

Integration: In-depth interviews revealed that the lack of integration is another challenge to using the EMRs as stated by the participants. The system administrator described this concern as follows:

‘There are some databases introduced by the non-governmental organizations and not integrated with the EMR. So, it would be good to be able to synchronize the databases with this EMR system. Integrating in one system can help us see all the patient information in one place at a point. This will help for monitoring and evaluation of service delivery within our health facility.’ (Age 34, HF3, Male, P6, System admin)

### Theme 4: Benefits of the electronic medical records

Participants expressed the benefits of the EMR besides reporting the challenges. They have shown the importance by comparing with the manual medical records. One of the benefits of the EMRs is easy information retrieval where health care workers can fetch their patients’ information in a moment from their office. This is incomparable with the paper record where a provider needs to contact the porter and may wait for an hour to get the patient’s medical chart.

Participants acknowledged this as:

‘The benefit is enormous. For example, if the patient’s information is lost in the paper record … I can easily find the patient’s medical history. I used to have a hard time finding patient information … but since I started using the electronic medical record, it becomes easy…’ (Age 32, Female, HF1, Nurse, P1)

‘Lab results used to be lost; now, lab test results that were done a long time ago are easily available.’ (Age 38, Female, HF4, Medical Laboratory, P7)

Another benefit reported by most of the participants was minimising duplication. This problem was seen in medical record numbers and patient charts. Before the implementation of the EMR, a single patient may have more than one medical record number and medical chart, which were documented for every visit. So, the EMR benefited the patient by having a unique record number.

This was supported by participants:

‘Prior to the introduction of the electronic medical record, there was a duplication of patient medical record numbers and duplication of patient charts. Now this is no longer a problem, a patient cannot have more than one medical record number with this system.’ (Age 35, Female, HF2, Medical Record P, P4)

‘[*I*]t helps to reduce the duplication of patients’ medical record number, because the patient record number is assigned by the system, so the same number is not assigned to more than one person. so, it can be able to provide us quality information with unique patient identifier.’ (Age 34, HF3, Male, P6, System admin)

## Discussion

In resource-limited countries such as Ethiopia, considering EMR for clinical care is no more a luxury. It is just becoming a requirement for providing modern standard care for patients and their families (Bisrat et al. 2021). Enhancing digital health technology is one of the strategic objectives of the Health Sector Transformation Plan II, aimed at addressing health system challenges and improving the coverage and quality of health practices and services (Ethiopian Ministry of Health 2021). In summary, the major barriers to the implementation of EMRs include inadequate EMR training, a lack of system comprehensiveness, poor interoperability, system-related issues, power interruptions and the absence of motivation schemes.

Electronic medical record training was compulsory for the effective implementation of the system in the health care facility. In this study, participants reported the gap in training by stressing that there was a need for EMR training for the effective implementation of the system and to have the ability to troubleshoot when there was a system failure or interruption. The finding was supported by many similar studies that assessed EMR training among health professionals, where the lack of technical training and support is the most common barrier to the implementation of EMR (Jimma & Enyew 2022; Yehualashet et al. 2021).

Ease of use can be assured with the comprehensiveness of a system, where all the necessary information and utilities for a specific task are found at one juncture. In this regard, there was a lack of comprehensiveness in that there were databases not integrated with the EMR system at some service units. In addition, clinical protocols, treatment guidelines and other supporting documents were not found in the system. Moreover, there were clinical and diagnostic terms not found in the system or with different terminologies. This is because of failure to adhere to the standard terminology. A qualitative study in the Russian Far East also reported poor adoption of standard terminology as a barrier to the implementation of the EMRs (Jung et al. 2020). In this study, comprehensiveness is also explained with the integration of information from different databases and health care providers into one seamless system. There were challenges in exchanging data between health care organisations and external entities such as insurances and finding the information in one seamless system (Jung et al. 2020).

Interoperability is another issue in the effective implementation of EMRs. It refers to the capacity of various information systems, devices and applications to access, exchange, integrate and collaboratively use data in a coordinated way across organisational, regional and national boundaries, ensuring timely and seamless information flow to enhance the health of individuals and populations worldwide (HIMSS 2022). Participants stated that the EMR system did not have the ability to communicate and exchange data with other systems from other health facilities. Hence, data integration and interoperability continue to be the most difficult challenges in health information systems. The EMRs are expected to connect different health facilities and services and ease the exchange of information. However, many of the participants felt that this was not fulfilled. This is because different health facilities have implemented EMRs with different infostructures, which hindered interoperability. As the EMR is implemented with a local area network, it cannot communicate with the system in other health facilities. This hinders access to patient information from any location. The interoperability issue appears to be a real challenge encountered because of a lack of coordination between health care facilities. Implementing interoperability standards is indeed vital for a successful countrywide EMR implementation. Understanding the power of partnership and promoting communication and coordination are required to attain an interoperable system (Oufkir & Oufkir 2023).

Funding organisations in collaboration with the Ministry of Health were engaged in piloting the EMR programme in a few health facilities. Most supporting organisations required the EMR programme to be sustainable after the partnership ceased. However, it will face difficulty continuing, as health facilities may not be prepared to take responsibility when the project ends. Participants explained that most of the support and assistance comes from non-governmental institutions and donor-funded projects, which did not ensure continuity. The study conducted in Morocco supported the previous statement, lack of taking or maintaining responsibilities. In the Moroccan study, the country’s digital health system was partially funded by various donors, including the European Union, the Middle-Income Countries Fund, the Multi-Donor Trust Fund for Countries in Transition and the World Bank. However, because of the diverse interests and priorities of EHR stakeholders, the implementation faced significant challenges (Oufkir & Oufkir 2023).

The qualitative study explained that the electric interruption was a significant obstacle to the success of EMR development. Sometimes, the power outage lasted for a day and occurred at an unpredictable time. This was one cause of the system slowdowns, and these system slowdowns were followed by many complaints of the health care workers practising the EMRs. Hence, data transport speeds become unstable and may cause the EMR to lag so much that screens could take up half an hour to load the system. This study finding is concurrent with a result from sub-Saharan Africa where the absence of strong health care facility infrastructure and electric power shortage limit the wider adoption of EMRs in sub-Saharan Africa (Femi Odekunle et al. 2018:13).

A reliable power source is vital for an effective and sustainable EMR system. Moreover, upgrading the central data repository or server was suggested. And, in case, if there were an incidental system interruption, it was recommended to develop a procedure to back up with a paper-based system and re-enter data from paper backups when the system was restored.

Most health care workers agreed on the need for acknowledgement and reward for celebrating the implementation achievements differently like certification or financial reward. In this study, participants believed that those who put in more time and effort ought to be rewarded financially or with a certification of some kind. Respondents stressed out that the job they do and the salary are not commensurate. This will decrease the motivation of the health care workers towards the use of the EMR. Even though there are contradictions on the sustainability of EMR implemented with motivation and reward schemes, most providers supported incentives and rewards for the development and acceptance of EMR. More studies supported this, where health professionals’ adoption, acceptance and attitude towards a new technology, such as an EMR system, may be influenced by individual motivation (Jedwab et al. 2022; Jung et al. 2020).

### Limitations

This study faced some limitations, like the budget constraints that prevented the inclusion of community or patient feedback on services received through the system. Language translation challenges also arose, as some native-language terms lacked direct English equivalents. Additionally, the self-administered data collection method, while cost-effective, flexible and private, posed risks of respondent misinterpretation with limited opportunities for clarification. To address the limitations, bilingual experts were engaged to ensure accurate translation of terms, and detailed instructions were provided to minimise misinterpretation in self-administered questionnaires. Furthermore, health care provider insights were used as proxies for patient feedback because of budget constraints.

### Implications or recommendations

To enhance the successful implementation of the EMR system, health care facilities should prioritise continuous professional development programmes to provide mandatory EMR training for both new and existing staff, ensuring they have the necessary skills for effective system use and troubleshooting. Additionally, policymakers and health care administrators should focus on improving system comprehensiveness and interoperability to facilitate seamless data exchange. Addressing infrastructure challenges, such as ensuring a reliable power supply and stable network connections, is crucial for system sustainability. Furthermore, introducing incentives and rewards for health care workers can enhance motivation and engagement in EMR adoption.

## Conclusion

In conclusion, the effective implementation of EMR systems is hindered by multiple interrelated challenges, such as limited system comprehensiveness, a lack of interoperability, unreliable power supply and absence of integrated databases, which undermine the usability and acceptance of EMRs among health care providers. Additionally, externally driven EMR projects often suffer from insufficient stakeholder engagement and ownership, affecting long-term sustainability. Technical limitations, such as server issues and a lack of backup systems, further weaken system resilience. Moreover, the lack of incentives and recognition for health care workers impedes motivation and engagement, particularly during early adoption phases. Addressing these challenges through comprehensive planning, stakeholder involvement, technical upgrades and motivation strategies is essential for optimising EMR performance and achieving intended health system improvements.
